# Breastfeeding and the risk of *rotavirus *diarrhea in hospitalized infants in Uganda: a matched case control study

**DOI:** 10.1186/1471-2431-11-17

**Published:** 2011-02-17

**Authors:** Eric Wobudeya, Hanifa Bachou, Charles K Karamagi, Joan N Kalyango, Edrisa Mutebi, Henry Wamani

**Affiliations:** 1 Department of Paediatrics & Child Health, Mulago National Referral Hospital. P. O. Box 7051 Kampala, Uganda; 2 Clinical Epidemiology unit, School of Medicine. Makerere University College of Health sciences. P. O. Box 7062 Kampala, Uganda; 3 Department of Internal Medicine, faculty of Medicine. Makerere University. P. O. Box 7062 Kampala, Uganda; 4 School of Public Health Makerere University College of Health Sciences. P. O. Box 7062 Kampala, Uganda

## Abstract

**Background:**

*Rotavirus *is responsible for over 25 million outpatient visits, over 2 million hospitalizations and 527,000 deaths annually, worldwide. It is estimated that breastfeeding in accordance with the World Health Organization recommendations would save 1.45 million children's lives each year in the developing countries. The few studies that examined the effect of breastfeeding on *rotavirus *diarrhea produced conflicting results. This study aimed to determine the effect of breastfeeding on *rotavirus *diarrhea among admitted infants in Uganda.

**Methods:**

The study was conducted in the Pediatrics medical emergency unit of a National Referral hospital during a peak incidence time for rotavirus from February to April 2008. It was an age matched case-control study with a ratio of 1:1. We consecutively enrolled infants presenting at the study site during this period whose caretakers consented to participate in the study. A minimum sample size of 90 pairs was adequate with power of 80% to detect a 30% decrease in breastfeeding rate among the cases assuming a breastfeeding rate of 80% in the controls. The infants with *rotavirus *positive results were the "cases". We used the commercial enzyme immunoassay kit (DAKO IDEIA™ rotavirus EIA detection kit) to diagnose the cases. The "controls" were admitted children with no diarrhea. We compared the cases and controls for antecedent breastfeeding patterns.

**Results:**

Ninety-one matched case-control age-matched pairs with an age caliper of one month were included in the analysis. Breastfeeding was not protective against rotavirus diarrhea (OR 1.08: 95% CI 0.52 - 2.25; p = 0.8) in the conditional logistic model.

**Conclusions:**

Our study findings did not reveal breastfeeding as protective against *rotavirus *diarrhea in infants. This suggests searching for other complementary preventive methods such as rotavirus vaccination and zinc supplementation to reduce the problem of *rotavirus *diarrhea in infants irrespective of their feeding practices.

## Background

Diarrhea is estimated to cause 1.5 million deaths and 21% of all under fives mortality worldwide [1]. It accounts for about five childhood deaths per 1000 population [2] mostly from developing countries. Annually *Rotavirus *diarrhea is associated with over 25 million outpatient visits, 2 million hospitalizations and 527,000 deaths per year worldwide [3]. Likewise, most of the under five diarrhea cases are caused by *rotavirus *diarrhea [4,5]. A study in Ghana found an incidence of 89 diarrhea episodes per 1000 children per year of which 35 episodes were due to rotavirus diarrhea [6].

The Human *rotavirus *infection spreads by direct person-to-person contact. Effective hand washing and disposal or disinfection of contaminated items is therefore theoretically an important measure in the prevention of *rotavirus *infection.

Breastfeeding could reduce gastrointestinal infections as breast milk contains lactadherine, secretory IgA, T & B-lymphocytes, bactericidal lactoferrin, oligosaccharides [7] and human milk glycans [8,9] that protect the intestinal epithelium against pathogens. The major component that is thought to prevent symptomatic rotavirus infection is lactadherine [10] while the anti-rotavirus antibodies in human milk seem to play a smaller role [11]. Although a study by Ray & Kelkar found low maternal serum neutralizing antibody titers to rotavirus in mothers whose children were recovering from severe rotavirus disease suggesting increased predisposition [12], another study by Asensi et al that quantified the human milk rotavirus IgA antibodies did not demonstrate this correlation [11]. Breast milk anti-bodies to rotavirus has however been sited as one of the possible factors for the low rotavirus vaccine efficacy seen in the low income countries compared to the high income countries [13]. The current WHO guidelines on diarrhea management recommend continued breastfeeding during the diarrhea episode.

However, the specific role of breastfeeding in the prevention of *rotavirus *diarrhea has not been well established but it is generally considered to at least the reduce the severity of the disease [14]. A case control study by Clemens J et al found that exclusive breastfeeding protects against rotavirus diarrhea in infants [15]. A prospective study by Naficy AB et al found a lower incidence of rotavirus diarrhea in infants that received breast milk [16]. A nested case control study by Dennehy PH et al found that breastfeeding in the previous month was protective in infants below 6 months [17]. Plenge-Bönig, A et al in a nested case control study in Europe of infants with acute gastroenteritis found breastfeeding to be protective against rotavirus gastroenteritis compared to other causes of gastroenteritis [18]. A review paper by Golding J et al did not find a protective effect of exclusive breastfeeding in infants aged 4 - 6 months against rotavirus diarrhea [19]. A prospective study by Misra SM et al found no difference in the rotavirus rates between the exclusively and non-exclusively breastfed infants [20]. The study may have not detected the difference because of the small number of 34 infants in the cohort studied. In a prospective study by Gurwith M et al breastfeeding appeared not protective against *rotavirus *diarrhea [21]. This study of 104 infants and there 62 siblings was not designed to determine the relationship between breastfeeding and rotavirus diarrhea. A longitudinal study by Linhares A et al in children aged 0 - 3 years in Brazil found no evidence of protection against clinical rotavirus disease by maternal milk [22].

The differences in study designs and age populations studied have led to variations in study results on effect of breastfeeding against *rotavirus *diarrhea. Therefore, we designed an age matched case-control study to investigate the effect of breastfeeding on *rotavirus *diarrhea among hospitalized Ugandan infants.

## Methods

### Study design

This was an age-matched case-control study with a ratio of 1:1. We matched controls within an age range of ± 1 month of the cases.

### Study setting

The study took place in the Pediatrics medical emergency unit of Mulago national referral hospital, Kampala, Uganda. This hospital also doubles as a primary health care centre for the surrounding areas. The Pediatric emergency unit of the hospital receives all children with severe medical conditions for overnight care. The children requiring further care transfer to one of the five main Pediatrics wards. The average monthly admission is about 1,000 children of whom about half of these are infants. About 7.1% of these infants have diarrhea with 45% due to *rotavirus *infection [23].

The definition of "Diarrhea" in this study was the passage of at least three loose or watery stools in any 24-hour period.

The cases consisted of infants admitted to the pediatric medical emergency unit of Mulago hospital with *rotavirus *diarrhea. The Controls were infants admitted to Pediatrics emergency unit of Mulago hospital with no diarrhea and had a negative stool sample for rotavirus. All parents/caregivers gave written informed consent to participate in the study. Caregivers are legal representatives of the participants other than the parents. We excluded infants with signs of respiratory tract infections and unknown feeding practices.

### Sampling procedure and matching

A trained research assistant screened all the infants with diarrhea reporting at the Pediatric emergency unit registration desk for the study. We consecutively enrolled into the study those fulfilling the eligibility criteria. All the eligible children had their stools collected within 24 hours of admission to avoid nosocomial rotavirus infection. We batched the stool samples for a maximum of 1 week at 8°C before rotavirus analysis. A trained research assistant to match the available cases screened children with no diarrhea reporting at the ACU registration desk for eligibility as admitted controls. The controls also had their stools collected within 24 hours of admission and analyzed for rotavirus within seven days. The recruitment of both the cases and controls was concurrent.

If more than one age-matched control was eligible for matching a case, we choose the control by simple random sampling from the available eligible controls. We wrote the controls' identification numbers on pieces of paper, and by random sampling, one paper chosen. The control corresponding to the chosen identification number was then age-matched with the available case. We captured the information from the parents/caregivers onto a standardized semi-structured questionnaire. All the children with diarrhea received standard of care for diarrhea that included rehydration therapy and zinc supplementation by the attending physicians.

### Measurements and data collection

The research assistant not aware of the *rotavirus *status of the infants collected information about feeding practices. The main feeding practice of interest was whether the infant was breastfeeding or not. We defined an infant as "breastfed" if breast milk, either received directly from the breast or expressed, constitutes any portion of the infant diet. Feeding practice recall period was limited to 1 week prior to the interview. The socio-demographic data collected included: infant's age in months, caregivers or parents' age, education level and occupation. We measured the infants' weight and length using a hanging Salter scale and a measuring board, respectively. We recorded the weight to the nearest 10 g and length to the nearest 10 cm. The research assistant inserted a size-8 feeding tube into the infant's rectum and used a 5 ml-syringe to aspirate at least three milliliters of the stool. A laboratory runner transferred the sample to the laboratory in a screw caped container within a maximum of 60 minutes of collection. A competent laboratory technologist trained in *rotavirus *identification tested the samples for *rotavirus *antigen using a commercial enzyme immunoassay kit (DAKO IDEIA™ rotavirus EIA detection kit) according to the standard operating procedures. The main outcome measure was the presence or absence of *rotavirus *antigens in the diarrhea stools of the study subjects.

### Data management and statistical analysis

We captured the data in EpiData v3.1 (The EpiData Association, Odense, Denmark) and analyzed using STATA v9.2 (Stata, College Station, TX, USA).

We used the computer-based command for sample size for matched case control studies using Stata version 9.2 to calculate sample size. A sample size of 90 cases and 90 matched controls was estimated to detect a thirty percent difference in the breastfeeding rates between the cases and controls assuming a prevalence of breastfeeding of 80% in the controls with an accepted type 1 error of 5% (α = 0.05 two-sided) and minimum power of 80%.

We carried out Bivariate analysis comparing the predictors and the outcome in the matched cases and controls using Mantel Haenszel method. We used backward conditional logistic regression to assess for independent predictors. The effect measure was matched odds ratio (conditional OR). We used Ninety-five percent test-based confidence intervals (CIs) for the odds ratio. In order to control for the extraneous variables on the relationship between breastfeeding and *rotavirus *diarrhea, we used the conditional logistic regression model. The level of statistical significance was p < 0.05.

### Ethical issues

We obtained Ethical approval from the Makerere university research and ethics committee before conducting the study. We obtained Written Informed consent from all the parents/caregivers.

## Results

### Description

The study took place in the Pediatrics medical emergency unit of a National Referral hospital during a peak incidence time for rotavirus from February to April 2008.

We screened two-hundred fifty (250) infants with acute diarrhea between February and April 2008. We excluded two infants due to unknown feeding practice and one hundred fifty seven had rotavirus negative diarrhea. Ninety-one stools were rotavirus positive. The 91 cases that were age-matched with the 91 controls were included in the analysis. The baseline characteristics between the cases and controls were similar except for the sex (see Table 1). The mean maternal age for the cases and controls was 24.2 (SD 5) and 24.4(SD 5.2) years respectively but this difference was not statistically significant (*p *= 0.7 student's t test).

**Table 1:** Some socio-demographic characteristics of 91 rotavirus cases and 91 controls in Kampala, Uganda

		Cases	Controls	*P *value
**Matching criteria**				
Age (months), mean (sd)		7.6(2.7)	7.5 (2.6)	0.86
**Characteristics**		**n (%)**	**n (%)**	
Sex	Female	27 (29.7)	41 (45.1)	0.032
	Male	64 (70.3)	50 (54.9)	
				
Wasting ^a^(Z score ≤-2)	Yes	15 (16.6)	16 (17.6)	0.87
	No	75 (83.3)	75 (82.4)	
				
Maternal education	Secondary & above	45 (49.4)	49 (53.8)	0.55
	Below secondary	46 (50.6)	42 (46.2)	
				
Maternal occupation ^a^	housewife	59 (66.3)	53 (58.8)	0.3
	others	30 (33.7)	37 (41.1)	
Crowding ^b^	Yes	28 (30.8)	25 (27.5)	0.62
	No	63 (69.2)	66 (72.5)	
Stool disposal	Improper	11 (12.1)	9 (9.9)	0.63
	Proper	80 (87.9)	82 (90.1)	

OR indicates odds ratio. CI indicates confidence interval

^a ^maternal occupation of 2 controls and 1 case were unknown; wasting data missing in 1 case

^b ^4 or more per room

The majority (70%) of our study infants were above the age of 6 months. The proportion of infants breastfeeding was 85% and 82% in the below 6 months and the above 6 months respectively. This difference was not statistically significant (p = 0.5). Among infants below 6 months, 77% of the cases and 92% of the controls were breastfeeding. This difference was not statistically significant (p = 0.1). Among infants above 6 months, 56% of the cases and 76% of the controls were breastfeeding. This difference was not statistically significant (p = 0.09). The breastfeeding rates were similar between the cases and the controls (see Table 2).

**Table 2:** Unadjusted association between some factors and *rotavirus *diarrhea in 91 matched case-control pairs in Kampala, Uganda

Variable	^**1**^**Cases (%)**	Controls (%)	^**2**^**OR**	95% CI	p-value
Crowding (4 or more per room)	31	27	1.1	0.6, 2.3	0.7
Sex (Male)	70	55	1.8	1.02, 3.4	0.04
^a ^Febrile (axilla T ≥ 37.5°C)	44	59	0.5	0.28, 1.003	0.05
^b ^Vomiting	85	35	20.5	4.9, 84.7	< 0.001
breastfeeding	85	81	1.2	0.5, 2.4	0.5
Complementary breastfeeding	66	59	1.3	0.7, 2.5	0.3
Exclusive breastfeeding	12	19	0.5	0.1, 1.3	0.2
Predominant breastfeeding	7	3	2	0.5, 7.9	0.5
^c ^Breastfeeding in Age > 6 Months	56	77	1.8	0.7, 4.4	0.15
^d ^Breastfeeding in Age = < 6 Months	77	92	0.3	0.06, 1.6	0.17
Improper disposal	12	10	1.2	0.4, 3.1	0.8
Wasted (≤ -2 Z)	17	18	0.9	0.4, 2.0	> 0.999
Secondary education and above	49	54	0.8	0.4, 1.5	0.6
Housewife	66	60	1.2	0.6, 2.3	0.5

^1^Proportion exposed to factor in case and control groups

^2^Odds ratio by Mantel-Haenszel method

^a ^85 pairs were analyzed. Not powered enough.

^b ^78 pairs were analyzed. This analysis variable is not powered enough.

^c ^70 pairs were analyzed. This analysis variable not powered enough.

^d ^20 pairs were analyzed. This analysis variable is not powered enough.

### Breastfeeding and rotavirus diarrhea

On matched bivariate analysis, breastfeeding was not associated with *rotavirus *diarrhea (Table 2). Complementary, exclusive and predominant breastfeeding were also not associated with *rotavirus *diarrhea (Table 2). However, this study did not have power to evaluate the various modes of breastfeeding.

The other factors associated with *rotavirus *diarrhea on matched bivariate analysis included vomiting, fever and sex (Table 2).

Sex and breastfeeding were included in the conditional logistic regression analysis. Vomiting and paternal education were not included because of significant missing data.

At multivariate analysis, using conditional logistic regression, breastfeeding was not protective OR 1.08 (95% CI 0.52 - 2.25) against *rotavirus *diarrhea in infants after controlling for sex (Table 3). There was no interaction between breastfeeding and other factors.

**Table 3:** Adjusted association between breastfeeding and *rotavirus *diarrhea in 91 matched case-control pairs in Kampala, Uganda

Variable	^**ξ**^**OR**	95% CI	p-value
Breastfeeding (yes)	1.08	0.52, 2.25	0.82
Sex (Male)	1.86	1.0, 3.42	0.048

^ξ^Odds ratio by Mantel-Haenszel method

## Discussion

This was a hospital based age-matched case-control study. The study was conducted on the assumption that breastfeeding is still being practiced in Uganda in accordance with cultural norms and recommendations from the WHO as an intervention to reduce the incidence and severity of diarrhea disease. However, its protective role against *rotavirus *is not universally accepted. The observation that the diarrhea rates and the median age of *rotavirus *disease are not delayed in countries with prolonged versus short durations of breastfeeding brings into question the protective role of breastfeeding against *rotavirus *diarrhea. This study aimed to investigate the relationship between breastfeeding and *rotavirus *diarrhea.

The results of this study did not demonstrate the protective effect of breastfeeding against *rotavirus *diarrhea in infants. Previous reports have concurred on this topic [21,24,25] in which none demonstrated significant overall protection of breastfeeding against *rotavirus *diarrhea. Duffy and Byers et al [14] followed a cohort of 197 infants through a winter season and found no difference in the rotavirus rates between the breast-fed and bottle-fed infants. Gurwith and Wenman et al [21] in a follow up study of 104 infants for 16.3 months found no difference in the rotavirus rates between the breastfed and non-breastfed infants. A nested matched case-control study by Weinberg et al [24] of 50 infants found no difference in the breastfeeding rates between infants with and without rotavirus diarrhea. An exploratory study by Glass et al [25] out of the surveillance data in Dhaka Bangladesh found higher rates of rotavirus among breastfed infants hence questioning the protective role of breastfeeding. In a case-control study conducted by Clemens et al [15] in Bangladesh with hospital cases and community controls, breastfeeding was not protective against *rotavirus *diarrhea in infancy. This study may have not had the power to detect this difference given the infrequency of non-breastfed infants in the studied population. The close linkage between age, breastfeeding and rotavirus diarrhea could probably explain the observations from these reports. The peak age for rotavirus diarrhea is 6-11 months while the rates of breastfeeding begin to decline after 6 months. The protective effects of breastfeeding seem to wane with age [15]. This might be the reason why findings from the studies where the focus was on infants of 6 months and below showed a tendency to protection by exclusive breastfeeding. In our study, the majority of the study participants were over 6 months of age. The differences in the methodologies and the various definitions of breastfeeding make the interpretation and comparisons of these studies less precise. The assumptions of our study may have not enabled us to detect the observed fifteen percent difference in the breastfeeding rates between the cases and the controls.

A sub-analysis report by Clemens et al focusing on infants of 6 months or less showed a strong association between breastfeeding and rotavirus diarrhea. This report is however not reliable given the very low rates of exclusively breastfed infants in the study. The exploratory analysis from our study showed a tendency to protection from rotavirus by exclusive breastfeeding mostly in infants below 6 months but was not powered enough to draw any conclusions. Dennehy, PH et al [17] showed a protective role of breastfeeding against hospitalization due to rotavirus diarrhea. Our study result may have differed because we measured current breastfeeding and not breastfeeding in the previous month, and our controls were hospitalized children. Plenge-Bonig, A et al [18] has showed a protective role of breastfeeding in infants with rotavirus acute gastroenteritis compared to other causes of gastroenteritis. Our study results may have differed from this work because none of our controls had diarrhea.

To our knowledge, there is scarcely any published data on the relationship between rotavirus and exclusive breastfeeding in infants less than 6 months.

The intestinal mucosa may need continuous bathing with antibodies and other anti-infective components in breast milk for protection against *rotavirus*. This implies that sporadic or low volume feeds may be ineffective. This observation has been made by Ebina [26] and Berger [27] where infants who were fed on appropriate volume of milk with *rotavirus *antibodies were protected or had reduced severity of *rotavirus *diarrhea. The possibility exists that breastfeeding may be protective only if it is practiced with the intensity and frequency that allows continuous high protection of the mucosa rather than the sporadic small volumes. Besides Hjelt K et al [28] showed that the levels of secretory IgA antibodies to *rotavirus *are highest in colostrums and early breast milk and rapidly decline in the first few weeks of life. The low vaccine efficacy found in Africa and Asia [29,30] might be explained by the early vaccination age and therefore the possibility of high vaccine interference by the higher levels breast milk antibodies [13].

In our study, sixty-three percent of the feeding practice was complementary breastfeeding. The factors determining the feeding practices are not random between the breastfed and non-breastfed infants. We may therefore be observing the impact of the complementary feeds on the risk of *rotavirus *diarrhea rather than the breastfeeding itself. Glass R et al [25] made a similar observation in the study where he observed an increased risk to *rotavirus *in infants 6-11 months and ninety percent of these children were on complementary breastfeeding. The observation in our study that none of the controls had a household contact with diarrhea supports the contagiousness of *rotavirus *within the home [31]. In our study, other factors such maternal education level, wasting in the infants and the methods of stool disposal did not influenced the relationship between breastfeeding and the risk of *rotavirus *diarrhea. The results from our study suggest that it may not just be breastfeeding but the mode of breastfeeding that determines its benefits. The protective effect of breastfeeding against *rotavirus *diarrhea has mainly been found in infants of 6 months and below [15]. These results also suggest that the observed non-benefit of breastfeeding against *rotavirus *may be due to environmental and sanitation factors rather than the breastfeeding itself. This therefore suggests that consideration of the background factors is critical in the realization of the benefits of breastfeeding on *rotavirus *diarrhea. Given the overall benefits of breastfeeding especially against bacterial diarrheal diseases, exclusive breastfeeding should be encouraged.

The study was limited by using hospital controls that usually are not representative of the community controls for the cases. The hospital controls are a highly selected group with unique and diverse background factors that do not represent the neighborhood factors among the cases. We however endeavored to limit this effect on our major outcome factor by excluding controls with conditions such as respiratory infections that are associated with breastfeeding. Our study design was unable to directly measure risk of rotavirus since the breastfeeding (exposure) and the outcome (rotavirus diarrhea) were measured at the same time. We reduced recall time for the feeding practice to one week in order to limit recall bias. If breastfeeding truly protects against only small doses of *rotavirus *diarrhea, then only cases with large inoculums may have developed severe disease requiring admission and therefore been included in our study. Our cases are a selected group of the severe forms of rotavirus infection. They may not be representative of the major pool of community rotavirus cases. We did age matching to cater for the differences in immunological responses and breastfeeding practice that change with age.

The study was about widely promoted concepts of breastfeeding and diarrhea in the public health messages. There is therefore the possibility that the responses from parents/caregivers were tailored to suit the health workers' expectations. We asked open questions concerning the feeding practices rather than whether the infants were breastfeed or not. We were however not able to verify the feeding practice in the infants. The majority of our study participants were above 6 months in whom the contribution of breastfeeding and supplemental feeding on rotavirus diarrhea could not be determined. If the intensity and frequency of breastfeeding influences the outcome of *rotavirus*, this study was not able to measure the intensities of breastfeeding between the cases and the controls.

We believe that these limitations did not significantly affect the results of this study.

## Conclusions

Our study findings failed to show breastfeeding as protective against *rotavirus *diarrhea in infants. Since most of our study participants were above six months of age and on complimentary feeding, we recommend another study particularly focusing on breastfeeding and *rotavirus *diarrhea in the first 6 months of life.

## Competing interests

The authors declare that they have no competing interests.

## Authors' contributions

WE: conceived the study design, contributed substantially to the acquisition of data, carried out the statistical analysis, interpreted the data and drafted the manuscript. BH: contributed to the study design and was involved in revising the manuscript. KKC: contributed to the refining of the study design, interpretation of results and drafting of the manuscript. NKJ: contributed to the study design and critical revision of the manuscript. ME: contributed to the study design and the manuscript revision. WH: participated in the study design and has been critically involved in revising the manuscript. All authors read and approved the final manuscript.

## Pre-publication history

The pre-publication history for this paper can be accessed here:

http://www.biomedcentral.com/1471-2431/11/17/prepub
